# Genome-Wide Characterization of bZIP Transcription Factors and Their Drought-Responsive Expression in *Astragalus membranaceus*

**DOI:** 10.3390/ijms27146275

**Published:** 2026-07-14

**Authors:** Jiemin Wang, Xiaoyuan Wang, Ye Zhang, Jiayao Chen, Lin Pei, Pei He, Huigai Sun, Xiaowei Han

**Affiliations:** 1 College of Pharmacy, Hebei University of Chinese Medicine, Shijiazhuang 050200, China; 2 Traditional Chinese Medicine Processing Technology Innovation Center of Hebei Province, Shijiazhuang 050200, China; 3 Hebei Academy of Traditional Chinese Medicine, Shijiazhuang 050031, China

**Keywords:** *Astragalus membranaceus*, bZIP transcription factors (TFs), codon usage bias, drought stress, expression analysis

## Abstract

*Astragalus membranaceus* is an important medicinal plant with considerable pharmacological and economic value; however, its growth and productivity are frequently threatened by drought stress. Basic leucine zipper (bZIP) transcription factors play crucial roles in plant growth, development, and abiotic stress responses, yet a comprehensive investigation of the bZIP gene family in *A. membranaceus* remains unavailable. In this study, 74 bZIP genes (*AmbZIPs*) were identified in the *A. membranaceus* genome and classified into 12 subfamilies based on phylogenetic relationships with *Arabidopsis thaliana*. Analyses of gene structure, conserved motifs, chromosomal distribution, and duplication events revealed high conservation within subfamilies and indicated that segmental duplication was the major driver of AmbZIP family expansion. Codon usage analysis showed that AmbZIP genes exhibited relatively weak codon usage bias, with codon preference predominantly shaped by natural selection rather than mutation pressure. A total of 23 optimal codons were identified, of which 91.3% were A/T-ending codons. Codon adaptability analysis further demonstrated that tobacco possessed the highest codon compatibility among five tested hosts, whereas Escherichia coli exhibited the lowest adaptability, suggesting that plant expression systems may be more suitable for functional studies of AmbZIP genes. Promoter analysis identified numerous cis-acting elements associated with phytohormone signaling and abiotic stress responses, particularly those related to abscisic acid, methyl jasmonate, salicylic acid, and drought responsiveness. Transcriptome analysis and quantitative real-time polymerase chain reaction (qRT-PCR) validation revealed that several AmbZIP genes were significantly induced under drought stress. Among them, *AmbZIP46* displayed strong drought-responsive expression, transcriptional activation activity, and exclusive nuclear localization. These findings provide the first comprehensive characterization of the bZIP gene family in *A. membranaceus* and establish a valuable foundation for elucidating drought-tolerance mechanisms and facilitating molecular breeding in this medicinal plant.

## 1. Introduction

Plants, as sessile organisms, are continuously exposed to fluctuating environmental conditions, including drought, high salinity, extreme temperatures, and other stresses associated with global climate change. Among these, drought represents one of the most severe constraints on plant growth, productivity, and geographical distribution. To cope with water deficit, plants have evolved sophisticated signal transduction networks that activate transcription factors (TFs), which in turn regulate downstream stress-responsive genes and reprogram cellular homeostasis. Therefore, transcription factors serve as central regulators in plant adaptation to environmental stress. Transcription factors are regulatory proteins that bind to specific cis-acting elements in the promoter regions of target genes, thereby activating or repressing transcription [1,2]. Numerous TF families have been reported to participate in plant responses to abiotic stresses [3,4], including bZIP (basic leucine zipper), MYB, NAC, and WRKY families. Among them, the bZIP family is one of the most evolutionarily conserved and functionally diverse TF families in eukaryotes [5,6]. Members of this family are widely involved in plant growth and development, hormone signaling, and responses to environmental stresses [7,8].

The characteristic feature of bZIP proteins is a highly conserved bZIP domain of approximately 60–80 amino acids, composed of two functional regions: a basic region responsible for DNA binding and nuclear localization, and a leucine zipper region mediating dimerization [9]. The basic region typically contains an N-X7-R/K motif that facilitates sequence-specific DNA recognition [10], while the leucine zipper consists of heptad repeats of hydrophobic residues that enable homo- or heterodimer formation [11]. This dimerization capacity expands the regulatory diversity of bZIP proteins and allows fine-tuning of transcriptional activation or repression.

Genome-wide analyses have identified bZIP gene families in numerous plant species. For example, 75 bZIP genes have been reported in *Arabidopsis thaliana* (*A. thaliana*) [12], whereas 61, 70, and 160 members were identified in *Artemisia annua* [13], *Salvia miltiorrhiza* [14] and *Glycine max* [15], respectively. Functional studies have demonstrated that many bZIP genes are closely associated with abiotic stress responses. Several bZIP members have been shown to enhance drought and salt tolerance through abscisic acid (ABA)-dependent signaling pathways, highlighting their crucial regulatory roles in stress adaptation [16,17,18,19]. Overexpression of *GmbZIP2* in *A. thaliana* and soybean increased plant resistance to drought and salt stresses [20]. One-third of the soybean *GmbZIP* genes were involved in regulating plant responses to abscisic acid (ABA), high salinity, drought, and low temperature [21]. The expression levels of rice *OsbZIP20* and *OsABA45* were significantly increased under drought stress conditions, and overexpression of *OsbZIP71* dramatically increased drought tolerance in rice [22]. Expression analysis of transcriptome data from six oil palm (*Elaeis guineensis*) tissues showed that 11 *EgbZIP* genes were highly expressed under cold, salt and drought stress conditions [23]. Overexpression of both pepper (*Capsicum annuum*) *CabZIP25* and wheat (*Triticum aestivum*) *TabZIP15* was able to increase their tolerance to salt stress [24,25]. *PpbZIP9* and *PpVIP1* are involved in cold shock-induced cold tolerance in peach fruit through effects on respiratory metabolism [26].

*Astragalus membranaceus* is a perennial medicinal herb belonging to the genus *Astragalus* (Fabaceae) and is widely cultivated in northern China. Its dried root, known as “Huangqi,” is extensively used in traditional medicine and contains diverse bioactive compounds such as saponins, flavonoids, and polysaccharides [27,28,29]. Beyond its medicinal importance, *A. membranaceus* also contributes to ecological restoration in arid and semi-arid regions due to its deep root system and drought adaptability. However, prolonged drought stress negatively affects its growth, yield, and active ingredient accumulation, posing challenges for large-scale cultivation.

With the recent release of the *A. membranaceus* reference genome [30], genome-wide investigations of gene families involved in stress responses have become feasible. Although transcriptomic studies have explored drought-responsive genes in this species, a comprehensive analysis of the bZIP transcription factor family has not yet been reported. Considering the pivotal role of bZIP TFs in abiotic stress signaling networks [31], a systematic characterization of this gene family is essential for understanding the molecular mechanisms underlying drought adaptation in *A. membranaceus*.

In this study, we conducted a genome-wide identification and characterization of the bZIP gene family in *A. membranaceus*. Phylogenetic relationships, chromosomal distribution, gene structure, conserved motifs, promoter cis-acting elements, and duplication events were comprehensively analyzed. Furthermore, transcriptome profiling and qRT-PCR validation were performed to investigate the expression patterns of AmbZIP genes under drought stress. Functional assays, including transcriptional activation in yeast and subcellular localization in *Nicotiana tabacum*, were conducted to explore the potential role of *AmbZIP46*. This work provides a genomic framework for understanding the evolution and stress-responsive functions of the bZIP family in *A. membranaceus* and offers candidate genes for future genetic improvement of drought tolerance.

## 2. Results

### 2.1. Identification and Phylogenetic Analysis of the bZIP Members in A. membranaceus

A total of 74 putative bZIP transcription factors were identified from the *A. membranaceus* genome and designated *AmbZIP1*–*AmbZIP74* according to their chromosomal positions (Table S1). The predicted AmbZIP proteins varied substantially in amino acid length, molecular weight, and isoelectric point. Protein lengths ranged from 132 to 693 amino acids, with predicted molecular weights ranging from 14.89 to 76.53 kDa. The theoretical pI values ranged from 4.72 to 10.13, indicating both acidic and basic proteins. Most AmbZIP proteins were predicted to be hydrophilic.

**Figure 1:**
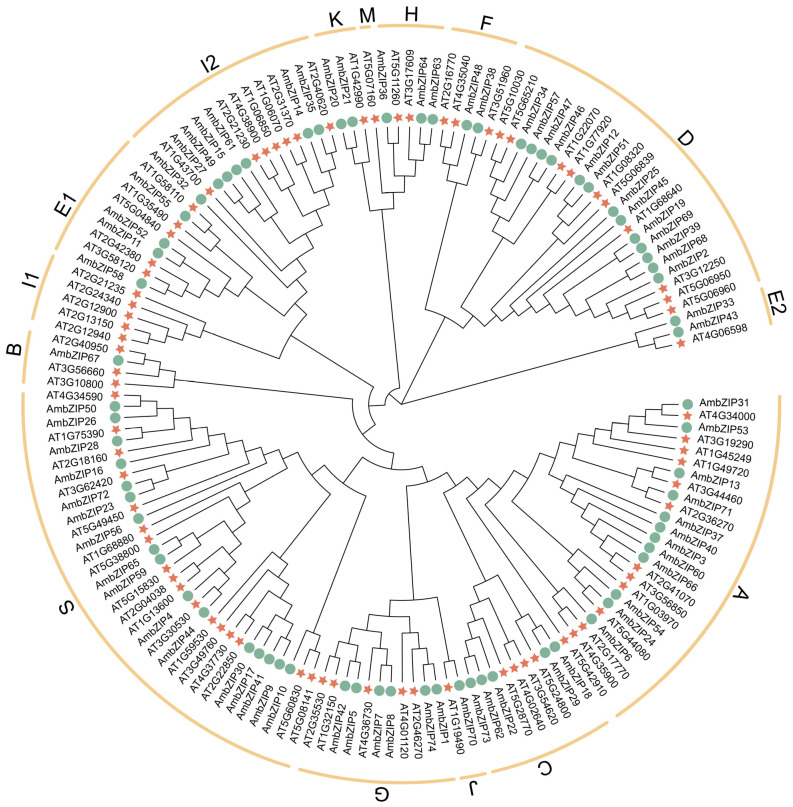
Phylogenetic analysis of the bZIP family TFs of *A. membranaceus* and *A. thaliana*. The phylogenetic tree of bZIP proteins was constructed using the Maximum Likelihood method with MEGA 11.0 software. Thirteen subgroups are indicated by different letters. The light green circle represents the bZIP family TFs of *A. membranaceus* and the orange red star pentagram represents bZIP family TFs of *A. thaliana*.

To investigate the evolutionary relationships of the AmbZIP family, a phylogenetic tree was constructed using full-length bZIP protein sequences from *A. membranaceus* and *Arabidopsis thaliana* (Figure 1). Based on the established classification system of Arabidopsis bZIP proteins, the AmbZIP members were classified into 12 subgroups, including A, B, C, D, E1, E2, F, G, H, I1, I2 and S. Subgroups A, D, and S contained relatively more members than other subgroups.

### 2.2. Conserved Domain, Gene Structure, and Motif Analysis of the bZIP Family in A. membranaceus

**Figure 2:**
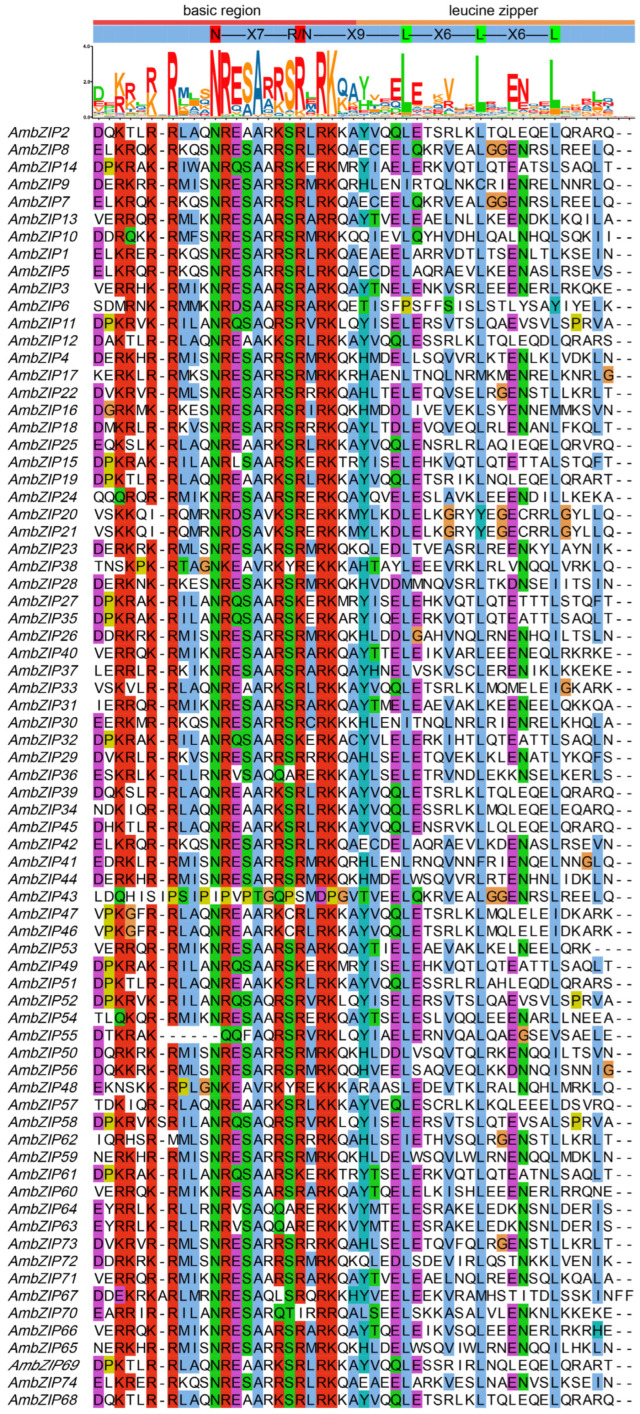
Multiple sequence alignment of the conserved domain of the bZIP proteins in *A. membranaceus*. The (**upper**) panel is a WebLogo 3 graph. The relative sizes of the letters indicate how often their corresponding amino acid appear at that position. The (**lower**) panel is a multiple sequence comparison of the 74 bZIP transcription factor members in *A. membranaceus*. Amino acids in the alignment are displayed in different colors according to their residue type, whereas in the WebLogo, the height of each letter is proportional to the frequency of the corresponding amino acid at that position.

Conserved domain analysis confirmed that all AmbZIP proteins contained the typical bZIP domain, including the conserved N-X7-R/K motif and leucine repeat regions (Figure 2). Several proteins also exhibited hydrophobic amino acid substitutions within the leucine zipper region.

**Figure 3:**
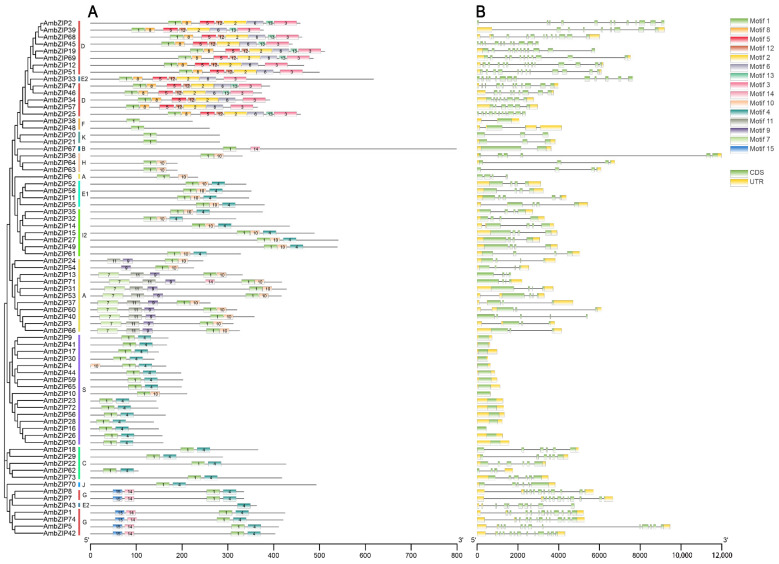
Motifs and gene structure of bZIP family in *A. membranaceus.* (**A**) Motif compositions of the bZIP proteins in *A. membranaceus.* The motifs are represented by rectangular boxes of different colors. (**B**) Identification of the *A. membranaceus* bZIP gene structure. Introns, CDS(s), and UTR(s) are represented by black lines, green rectangles, and yellow rectangles, respectively.

Motif analysis identified multiple conserved motifs among AmbZIP proteins (Figure 3). Motif 1 was present in nearly all members and corresponded to the conserved bZIP domain. Several motifs displayed subgroup-specific distribution patterns. Members within the same phylogenetic subgroup generally shared similar motif compositions.

Gene structure analysis showed substantial variation in exon–intron organization among AmbZIP genes. The number of exons ranged from 1 to 12. Genes belonging to the same subgroup generally exhibited similar exon–intron structures, whereas marked differences were observed among different subgroups. These results indicate structural conservation within subgroups and diversification among different clades.

### 2.3. Chromosomal Distribution and Duplication Analysis of the bZIP Family in A. membranaceus

**Figure 4:**
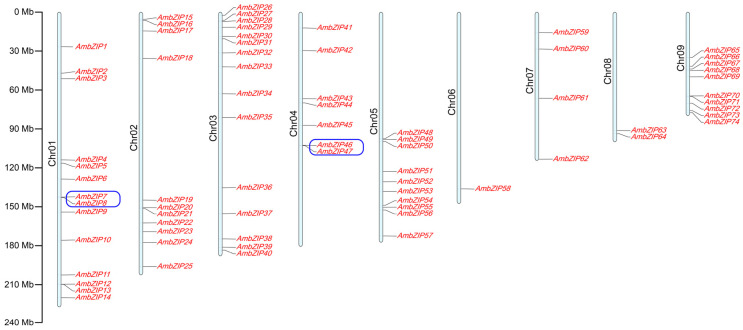
Chromosomal distribution of the bZIP transcription factor family genes in *A. membranaceus*. The blue circles indicate the tandem-duplicated bZIP genes.

**Figure 5:**
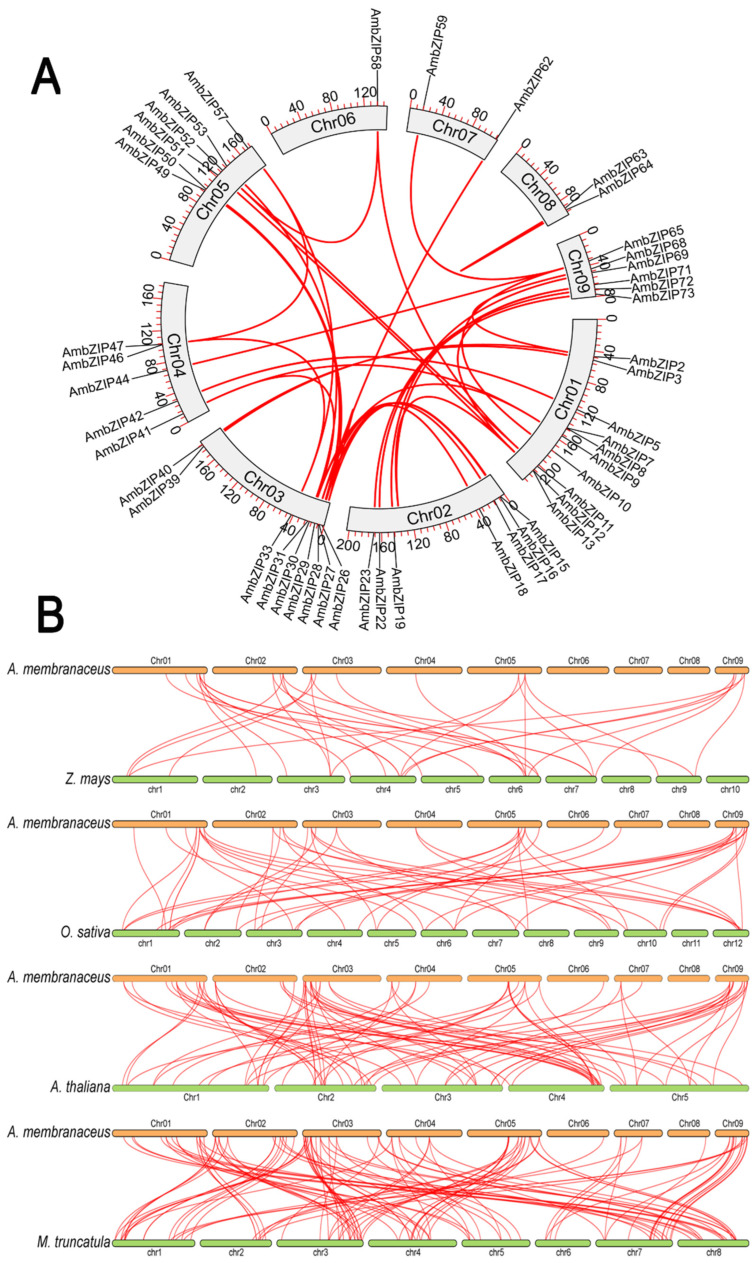
Segmental duplication and synteny analysis of the bZIP genes in *A. membranaceus*. (**A**) The distribution of segmental duplicated bZIP gene on *A. membranaceus* chromosomes. (**B**) Collinearity analysis of the bZIP genes of *A. membranaceus* with those of *Z. mays*, *O. sativa*, *A. thaliana*, and *M. truncatula*. The red lines in the figure represent syntenic relationships (collinearity) between bZIP genes of A. membranaceus and those of other species (including Z. mays, O. sativa, A. thaliana, and M. truncatula). These connecting lines visually demonstrate the homologous relationships and genomic collinearity of bZIP genes across different species at the genome-wide level.

To investigate the evolutionary expansion of the AmbZIP family, tandem and segmental duplication events were further analyzed across the *A. membranaceus* genome. A total of two tandemly duplicated gene pairs (AmbZIP7/AmbZIP8 and AmbZIP46/AmbZIP47) and 33 segmentally duplicated gene pairs were identified (Figure 4 and Figure 5A), indicating that segmental duplication represented the major expansion pattern of the AmbZIP family, whereas tandem duplication contributed to a lesser extent.

Comparative synteny analysis revealed extensive collinear relationships between *A. membranaceus* and other plant species (Figure 5B). More homologous bZIP gene pairs were identified between *A. membranaceus* and *Medicago truncatula* than between *A. membranaceus* and the other examined species, indicating a relatively closer evolutionary relationship between the two leguminous plants.

To evaluate the evolutionary selection pressure acting on duplicated AmbZIP genes, the nonsynonymous substitution rate (Ka) and synonymous substitution rate (Ks) of paralogous gene pairs were calculated (Table S2). All duplicated gene pairs exhibited Ka/Ks ratios lower than 1, indicating that purifying selection predominated during the evolution of the AmbZIP gene family.

### 2.4. Codon Usage Bias and Evolutionary Constraint Analysis of the bZIP Family Genes in A. membranaceus

To characterize codon usage patterns of AmbZIP genes, nucleotide composition and codon usage indices were analyzed. The GC contents at the first and second codon positions were generally higher than those at the third codon position. Most AmbZIP genes exhibited relatively weak codon usage bias, with ENC values mainly distributed above 45.

**Figure 6:**
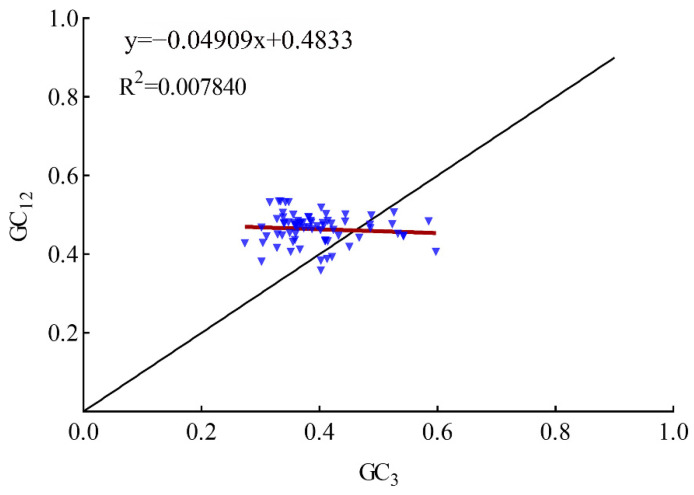
Neutrality plot analysis. The diagonal black line indicates GC_3_ = GC_12_ (y = x reference line). Deviations from this line suggest the influence of natural selection on codon usage bias.

**Figure 7:**
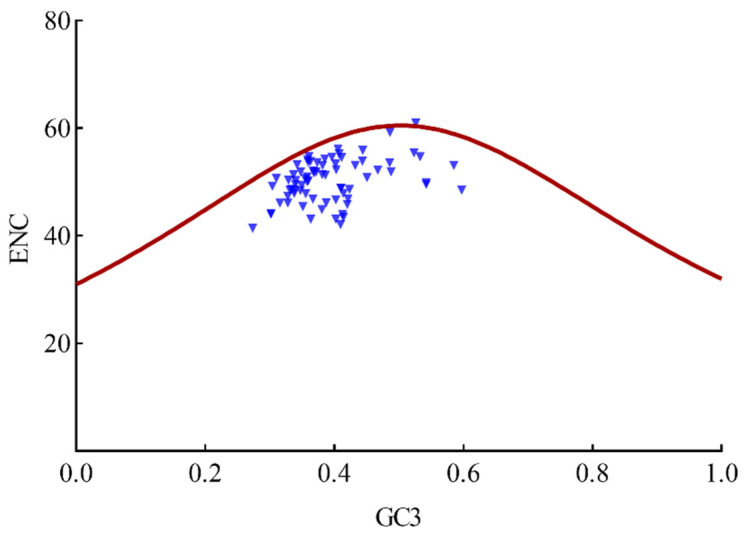
ENC-plot analysis. The red curve indicates the expected ENC values when codon usage is random (determined solely by GC_3_ content). Deviation below the curve suggests the influence of natural selection.

**Figure 8:**
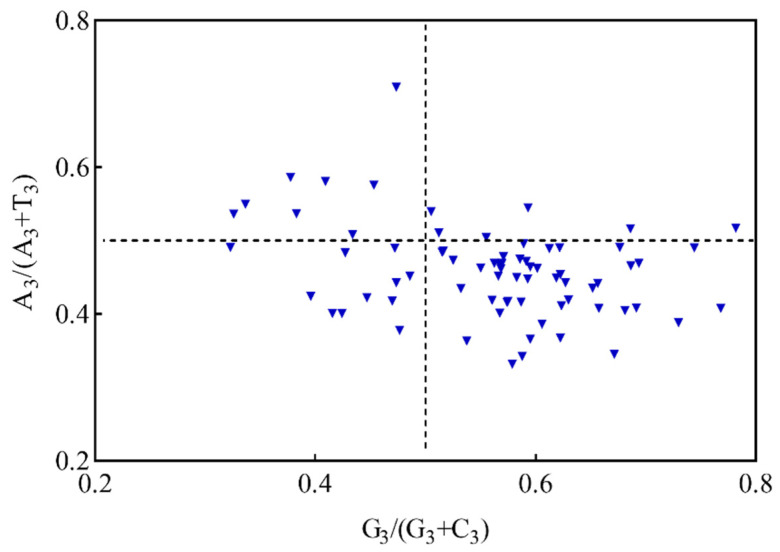
Codon usage bias analysis.

Neutrality plot analysis showed a weak correlation between GC12 and GC3 (Figure 6), indicating a limited contribution of mutation pressure to codon usage variation. In the ENC plot, most genes were distributed below the expected curve (Figure 7), suggesting that codon usage was influenced by factors other than nucleotide composition. PR2-bias analysis showed unequal usage frequencies of A/T and G/C at the third codon position (Figure 8).

A total of 23 optimal codons were identified in the AmbZIP gene family, among which A/T-ending codons predominated. Correlation analysis revealed significant associations among GC content, ENC, CAI, CBI, and FOP values (Table S3).

### 2.5. Analysis of Optimal Codon Usage and Heterologous Expression Adaptability of the bZIP Family Genes in A. membranaceus

Based on the codon usage bias analyses described above, the optimal codons of the *A. membranaceus* bZIP gene family were further examined to elucidate patterns of synonymous codon preference (Table S4). A total of 23 optimal codons were identified within the bZIP gene family, including AAT, ACA, ACT, AGA, AGG, AGT, CAA, CAT, CCA, CCT, CTT, GAA, GAT, GCA, GCT, GGA, GGT, GTG, TCA, TCT, TGT, TTA, and TTT. Among these optimal codons, 22 ended with A/T, accounting for 91.30% of the total, whereas only two codons (AGG and GTG) ended with G/C, representing 8.70%. The marked predominance of A/T-ending optimal codons is consistent with the nucleotide composition and codon usage patterns observed in previous analyses, indicating a strong bias toward A/T-ending synonymous codons in the *A. membranaceus* bZIP gene family.

The blue triangles represent AmbZIP genes in the PR2-bias plot. The dashed lines at x = 0.5 and y = 0.5 indicate unbiased usage of A/T and G/C at the third codon position, respectively. Their intersection (0.5, 0.5) represents no codon usage bias; deviations from this center suggest the presence of bias.To evaluate the codon adaptability of the *A. membranaceus* bZIP gene family in heterologous expression systems, the ratios of codon usage frequencies (Sc/host) between the coding sequences of bZIP genes and five representative hosts, including *Nicotiana tabacum*, *Arabidopsis thaliana*, *Solanum lycopersicum*, *Saccharomyces cerevisiae*, and *Escherichia coli*, were systematically analyzed. Using 0.5–2.0 as the acceptable range for codon adaptability (Table S5), statistical analysis showed that 62, 55, and 59 codons in tobacco, Arabidopsis, and tomato, respectively, fell within this adaptive interval, whereas yeast and *E. coli* contained 57 and 46 adaptable codons, respectively. These results indicate that tobacco exhibits the greatest codon compatibility among the five hosts, suggesting that it may serve as the most suitable plant recipient system for heterologous expression of the *A. membranaceus* bZIP gene family. In contrast, *E. coli* displayed the lowest level of codon adaptability, implying that this prokaryotic expression system may face substantial codon usage bias barriers during functional expression and therefore require codon optimization. Collectively, these findings suggest that the codon usage patterns of the *A. membranaceus* bZIP gene family show greater evolutionary concordance with eukaryotic hosts, particularly higher dicotyledonous plants.

### 2.6. Analysis of Cis-Acting Elements of the bZIP Family Genes in A. membranaceus

**Figure 9:**
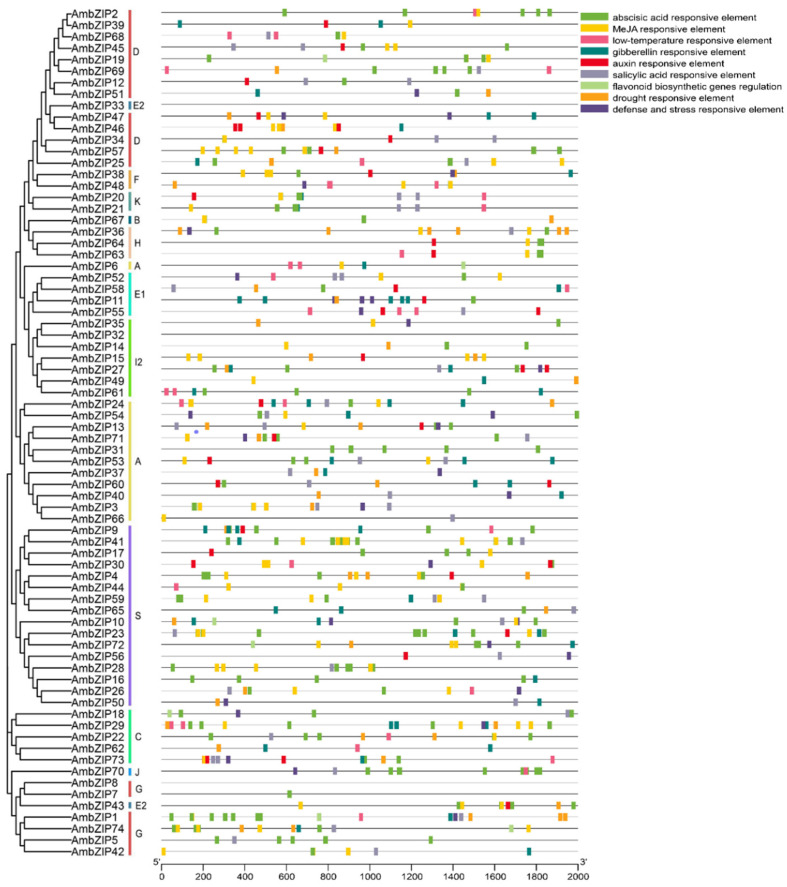
Analysis of cis-acting elements in the promoter regions of the *A. membranaceus* bZIP genes. Different types of cis-acting elements are visually represented by different colored boxes. The positions of cis-acting elements in the promoter regions can be estimated by the scale at the bottom.

The 2000 bp upstream promoter sequences of all AmbZIP genes were analyzed using the PlantCARE database to identify cis-acting elements associated with hormone signaling and stress responses (Figure 9). A large number of hormone- and stress-responsive cis-elements were identified in the promoter regions of AmbZIP genes. More than half of the AmbZIP promoters contained abscisic acid (ABA)-responsive, methyl jasmonate (MeJA)-responsive, and salicylic acid (SA)-responsive elements, indicating that ABA-, MeJA-, and SA-related regulatory elements are widely distributed within the AmbZIP family. In addition, gibberellin-responsive and drought-responsive elements were detected in the promoters of 35 and 34 AmbZIP genes, respectively. By contrast, auxin-responsive, defense- and stress-responsive, and low-temperature-responsive elements occurred in relatively few promoters. No stress- or hormone-related cis-elements were identified in the promoter regions of AmbZIP8, AmbZIP32, or AmbZIP33. Furthermore, flavonoid biosynthesis-related cis-elements were detected in eight AmbZIP genes. These results suggest functional diversification of AmbZIP genes in hormone signaling, stress responses, and secondary metabolism.

### 2.7. Expression Profiling of the bZIP Family Genes in A. membranaceus Based on the Transcriptome Data

**Figure 10:**
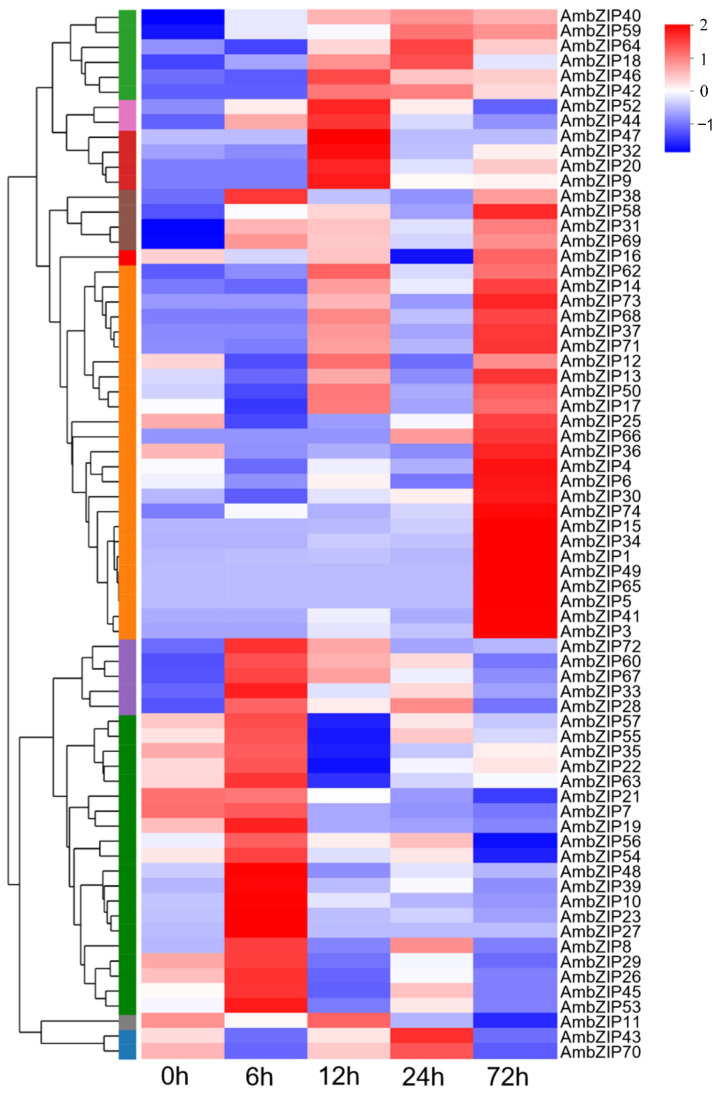
Hierarchical clustering analysis of bZIP transcription factor expression profiles under drought stress at 0, 6, 12, 24, and 72 h. Red and blue colors indicate relatively high and low expression levels, respectively. Genes with similar color patterns across time points are clustered together in the same branch, indicating comparable expression trends.

To characterize the expression changes of bZIP transcription factors under drought stress, hierarchical clustering analysis was performed using transcriptome data from five time points (0, 6, 12, 24, and 72 h) (Figure 10). The results revealed distinct expression patterns among family members, which could be broadly divided into two major categories. The first category (e.g., *AmbZIP39*, *AmbZIP48*, and *AmbZIP54*) exhibited an initial increase followed by a decrease, with peak expression at 6 h. The second category (e.g., *AmbZIP30*, *AmbZIP58*, and *AmbZIP74*) showed a gradually increasing trend, reaching the highest expression level at 72 h. Meanwhile, several genes, including *AmbZIP44* and *AmbZIP47*, displayed transient expression peaks within the 12–24 h window, suggesting their potential involvement in the mid-phase regulation of stress responses. Collectively, these findings reveal the highly heterogeneous temporal expression strategies of bZIP genes in *A. membranaceus* in response to drought, reflecting their potentially diverse biological functions.

### 2.8. qRT-PCR Analysis of Expression of 16 bZIP Genes in A. membranaceus Under Drought Stress

**Figure 11:**
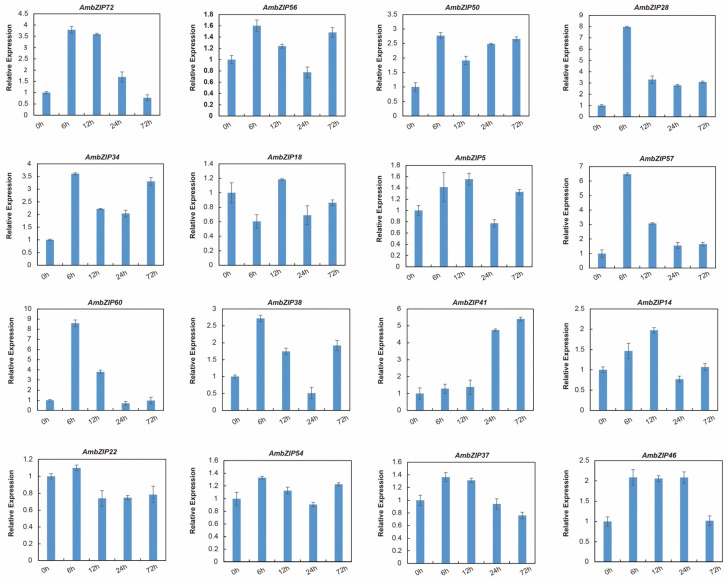
qRT-PCR analysis of the AmbZIP genes under drought stress. The ordinate is the relative expression calculated by 2^−ΔΔCt^. The horizontal coordinate is the time points of drought stress treatment. Three replicates are performed for each sample, and the error bar represents the standard deviation between the three replicates.

To further validate the transcriptional responses of bZIP family members to drought stress, qRT-PCR analysis was performed on 16 selected bZIP genes in *A. membranaceus.* As illustrated in Figure 11, dynamic and distinct expression profiles were observed throughout the 72 h drought treatment. Among the examined genes, nine bZIP genes, including AmbZIP72 and AmbZIP41 (which showed the most prominent induction among the tested genes), exhibited significant transcriptional induction, with expression levels increasing by more than twofold under drought stress. In contrast, none of the analyzed genes showed a reduction exceeding twofold, suggesting that drought stress predominantly activated rather than repressed bZIP gene expression in *A. membranaceus*.

Notably, the drought-responsive bZIP genes displayed divergent temporal expression patterns. The expression of AmbZIP41 increased progressively throughout the stress period and reached its maximum level at 72 h, implying a potential role in late-stage drought adaptation. By comparison, the remaining eight drought-induced genes exhibited rapid transcriptional activation and peaked at the early stress stage (6 h), followed by varying degrees of decline or stabilization during prolonged stress exposure. These results indicate that bZIP transcription factors participate in distinct phases of the drought response and may function coordinately in regulating stress adaptation in *A. membranaceus.* The amplification efficiency for each primer pair ranged from 92% to 108% (Table S6), falling within the acceptable range recommended by the MIQE guidelines.

### 2.9. Transcriptional Activation Activity and Subcellular Localization of AmbZIP46

To further elucidate the functional characteristics of drought-induced bZIP genes, *AmbZIP46* was selected as a representative candidate for transcriptional activation and subcellular localization analyses.

**Figure 12:**
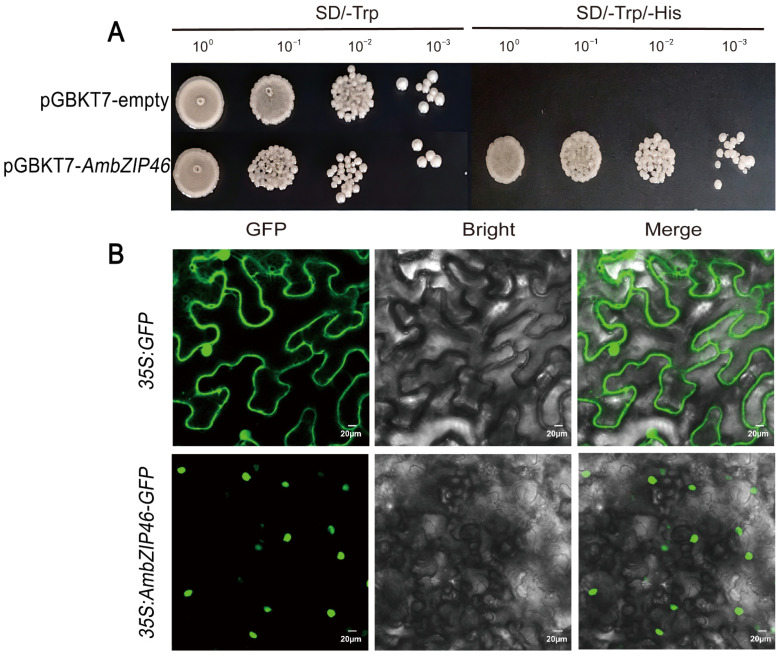
Transcriptional activation activity and subcellular localization of AmbZIP46. (**A**) Assay of transcriptional activation activity of *AmbZIP46*. SD/-Trp and SD/-Trp/-His are single- and double-deficient media, respectively. (**B**) Subcellular localization analysis of AmbZIP46 in tobacco leaf cells. 35S:GFP (empty vector) was used as a control. GFP: Fluorescent channel. Bright: Bright field. Merge: Colocalization image.

The transcriptional activation activity was evaluated using a yeast one-hybrid system. As shown in Figure 12A, yeast cells harboring the recombinant vector *pGBKT7-AmbZIP46* were able to grow normally on both selective medium lacking tryptophan (-Trp) and double-deficient medium lacking tryptophan and histidine (-Trp/-His). In contrast, yeast cells transformed with the empty vector *pGBKT7* failed to grow on the double-deficient medium. These results indicate that AmbZIP46 possesses intrinsic transcriptional activation activity.

For subcellular localization analysis, the AmbZIP46-GFP fusion construct was transiently expressed in tobacco (*Nicotiana benthamiana*) leaf epidermal cells. As shown in Figure 12B, the GFP signal of the negative control (35S::GFP) was distributed in both the nucleus and cytoplasmic intercellular regions, whereas the fluorescence signal of AmbZIP46-GFP was predominantly accumulated in the nuclear region, suggesting that AmbZIP46 is a nuclear-localized protein.

## 3. Discussion

bZIP transcription factors represent one of the largest and most evolutionarily conserved families of transcriptional regulators in plants, playing central roles in growth and development, hormone signaling, and responses to abiotic stresses. In this study, 74 bZIP genes were identified in the genome of *A. membranaceus* [32]. This number is comparable to that reported in several other plant species, including *Arabidopsis thaliana*, rice, and *Glycyrrhiza uralensis* [12,22,33], suggesting that the bZIP family has maintained a relatively stable expansion pattern across angiosperms, potentially shaped by species-specific selective pressures.

Phylogenetic analysis classified the AmbZIP proteins into 10 well-conserved subgroups and further aligned them with the established classification system in Arabidopsis, including subgroups A–I, S, and additional groups J, K, and M [34,35]. This indicates that the bZIP family has undergone long-term evolutionary conservation of its functional framework. While members within the same subgroup exhibited highly similar gene structures and conserved motif compositions, marked differences were observed among distinct subgroups, highlighting the coexistence of structural conservation and functional diversification within this gene family. Notably, several subgroups (such as S, H, and K) contained fewer introns or were intronless, which may facilitate rapid transcriptional responses under stress conditions by reducing RNA processing time [36,37,38].

Gene duplication analysis revealed that segmental duplication served as the predominant mechanism driving the expansion of the AmbZIP family, whereas tandem duplication played a relatively minor role. This pattern is consistent with observations in other leguminous and higher plant species [39,40,41]. Furthermore, all duplicated gene pairs exhibited Ka/Ks ratios below 1, indicating strong purifying selection and suggesting functional constraint throughout evolution. Synteny analysis further demonstrated a higher number of orthologous gene pairs between *A. membranaceus* and *Medicago truncatula* as well as *Arabidopsis thaliana*, reflecting their closer evolutionary relationships and the conservation of bZIP functional modules within dicot species.

Promoter analysis revealed that AmbZIP genes are enriched in cis-acting elements associated with ABA, MeJA, and SA signaling, along with numerous drought-responsive elements. This suggests that the bZIP gene family is widely involved in complex hormone- and stress-regulatory networks. The widespread presence of ABA-responsive elements further supports a central role for bZIP genes in drought signaling pathways, given the well-established function of ABA in water-deficit responses. In addition, several genes contained cis-elements related to flavonoid biosynthesis, implying that bZIP transcription factors may also regulate secondary metabolic pathways. Since flavonoids contribute to antioxidant defense and stress tolerance, bZIP genes may integrate stress signaling with metabolic regulation.

At the molecular evolution level, codon usage bias analysis indicated that AmbZIP genes exhibit relatively weak codon preference, characterized by high ENC values and a predominance of A/T-ending codons. These patterns are consistent with general codon usage features observed in dicot genomes. Neutrality plot, ENC-plot, and PR2 analyses collectively demonstrated that natural selection plays a dominant role in shaping codon usage patterns, while mutational pressure has a relatively limited contribution. This suggests that AmbZIP genes are under functional constraints that help maintain stable translational efficiency and regulatory consistency.

Further analysis of optimal codons revealed species-specific codon preferences within the AmbZIP family, which may be associated with translational optimization. Heterologous expression adaptability analysis showed that *Nicotiana tabacum* exhibited the highest codon compatibility with AmbZIP genes, whereas *Escherichia coli* showed the lowest compatibility. These findings indicate that AmbZIP genes are more evolutionarily adapted to eukaryotic expression systems, particularly dicotyledonous plants, providing useful guidance for future functional characterization and transgenic expression strategies.

Expression profiling revealed that AmbZIP genes exhibit pronounced temporal divergence under drought stress, and can be categorized into constitutively high-expression genes, early-responsive genes, and dynamically regulated genes. This expression heterogeneity suggests that different bZIP members function at distinct stages of drought response, forming a coordinated regulatory cascade from early stress perception to downstream adaptive responses.

qRT-PCR validation further confirmed the reliability of transcriptome data, with most tested genes showing upregulated expression under drought conditions. This indicates that AmbZIP genes generally act as positive regulators in stress responses. Among them, *AmbZIP41* showed sustained induction, suggesting a potential role in long-term stress adaptation, whereas other genes were mainly involved in early rapid responses, further supporting functional divergence within the family.

Functional characterization demonstrated that *AmbZIP46* possesses transcriptional activation activity and is localized in the nucleus, consistent with the canonical features of transcription factors. Combined with its drought-inducible expression pattern and regulatory potential, *AmbZIP46* is likely to function as a positive regulator within the ABA-mediated drought response network. This gene was selected for functional analysis based on its close phylogenetic relationship with the well-characterized drought-responsive *OsbZIP46* in rice and its significant drought-inducible expression (|log_2_FC| > 2, FDR < 0.01) in our transcriptome data [1]. Based on the expression profiles, we hypothesize that different AmbZIP members may function collectively at distinct phases of drought responses, forming a coordinated regulatory cascade. Specifically, subgroup A and D members (e.g., *AmbZIP46*) were rapidly induced at the early stage (6–12 h), likely acting through ABA-dependent signaling to initiate rapid stress responses, whereas subgroup S members (e.g., *AmbZIP41*) exhibited sustained upregulation during prolonged stress (24–72 h), suggesting roles in long-term adaptive processes. Among these, *AmbZIP46* represents the most promising candidate for enhancing drought resilience, while *AmbZIP41* may also contribute to long-term adaptation. However, transgenic functional validation is still required to confirm these hypotheses [13,14].

However, we acknowledge that DAPI nuclear staining was not included as an independent marker in our subcellular localization assay, which represents a limitation; future studies will incorporate additional approaches to further strengthen this conclusion. Additionally, the seedlings were derived from seeds rather than from inbred lines, and we acknowledge that genetic heterogeneity cannot be completely excluded given the perennial nature of *A. membranaceus*. Future studies should consider using inbred lines or clonally propagated materials to minimize this potential effect.

## 4. Materials and Methods

### 4.1. Plant Materials and Stress Treatment

Our research complies with all relevant institutional, national, and international guidelines and regulations. The collection of *A. membranaceus* was conducted under appropriate permits/licenses. The seeds of *A. membranaceus* were collected from Dingxi county, Gansu province, China (geographical coordinates: 35°58′ N, 104°63′ E). With formal permission from the administrators of the Medicinal Plant Garden at Hebei University of Chinese Medicine (HBUCM), the seeds were introduced and cultivated in the Medicinal Plant Garden on the HBUCM campus (No. 3 Xingyuan Road, Luquan District, Shijiazhuang City, Hebei Province, China; 38°05′ N, 114°34′ E). All experimental seeds and plant materials used for the drought-stress treatments were uniformly harvested from this authorized experimental plot once the plants reached maturity. The sampling activities were explicitly approved by the management of the Medicinal Plant Garden at HBUCM. Voucher specimens of the seeds (Specimen No.: 2024101501001LY) and seedlings (Specimen No.: 2025061701002LY) have been deposited in the Herbarium of Hebei University of Chinese Medicine. Two-month-old-seedlings with similar growth statuses were used for PEG6000 simulated drought stress treatment. All seedlings were randomly divided into 6 groups, and one group was grown in normal conditions and was used as the control group. The four drought stress treatment groups were irrigated with 20% PEG6000 for 6 h, 12 h, 24 h, and 72 h. During the PEG treatment, soil moisture was monitored daily using a ZY1200 portable soil moisture meter (manufactured by China Electronic Technology Information Industry Co., Ltd. (Zhengzhou, Henan, China); measuring range: 0–1.0 m^3^·m^−3^; accuracy: ±0.02 m^3^·m^−3^), and the soil relative water content was maintained at 35–40% of field capacity. Samples from the control and treatment groups were collected, frozen in liquid nitrogen and stored at −80 °C until RNA extraction. This was repeated three times for each sampling time point. Three biological replicates were taken for each sampling time point, and each biological replicate consisted of pooled leaf samples from at least three seedlings.

### 4.2. Identification and Physicochemical Property Analysis of the bZIP TF Family in A. membranaceus

The genome data of *A. membranaceus* were downloaded from the Global Pharmacopeia Genome Database (GPGD) (http://www.gpgenome.com/species/109, accessed on 15 April 2024). To systematically identify bZIP transcription factors, we employed a multi-step validation approach. The Hidden Markov Model profiles for the bZIP TF family (PF00170 and PF07716) were acquired from the Pfam database (http://pfam.xfam.org/) and implemented using HMMER 3.3.0 software (http://hmmer.janelia.org/, accessed on 15 April 2024) for initial screening against the *A. membranaceus* genome.

The predicted candidates were further validated through NCBI Batch CD-Search (https://www.ncbi.nlm.nih.gov/cdd, accessed on 20 April 2024) to confirm the presence of complete bZIP domains, while SMART (https://smart.embl.de/, accessed on 20 April 2024) analysis was used to verify the conserved basic region and leucine zipper motifs. For additional confirmation, we performed BLASTP alignment against the reference bZIP protein sequences from *Arabidopsis thaliana* (downloaded from TAIR, https://www.arabidopsis.org/, accessed on 20 April 2024) using a threshold of e-value ≤ 1 × 10^−5^ and coverage ≥ 50%.

Only genes consistently identified by both HMM search and BLAST alignment were retained as high-confidence bZIP members. A total of 74 non-redundant bZIP genes were obtained and systematically renamed as AmbZIP1–AmbZIP74 according to their chromosomal positions. Comprehensive physicochemical characterization, including protein length, molecular weight, isoelectric point, and other indicators, was conducted using the ProtParam tool (https://web.expasy.org/protparam/, accessed on 25 April 2024) [42,43].

### 4.3. Multiple Sequence Alignment and Phylogenetic Analysis of the bZIP Gene Family in A. membranaceus

Multiple sequence alignment was carried out using DNAMAN 6.0 (https://www.lynnon.com/dnaman.html, accessed on 12 May 2024) and ClustalW (https://www.genome.jp/tools-bin/clustalw, accessed on 20 May 2024). The amino acid sequences of *A. thaliana* bZIP proteins were downloaded from the *A. thaliana* information resource (TAIR, https://www.arabidopsis.org/) [12]. The best-fit amino acid substitution model was determined using the “Find Best Protein Model (ML)” function in MEGA 11.0, and the LG (Le_Gascuel_2008) model was selected based on the lowest Bayesian Information Criterion (BIC) and corrected Akaike Information Criterion (AICc) values. The phylogenetic tree was constructed with MEGA 11.0 software (https://megasoftware.net/) using the Maximum Likelihood method based on the LG model, and the bootstrap value was set to 1000 replicates to assess node support [44].

### 4.4. Motif Prediction, Gene Structure and Promoter Analysis of the bZIP TF Family in A. membranaceus

The conserved motifs of the bZIP proteins were identified using Multiple Expectation Maximization for Motif Elicitation (MEME) 4.11.4 (http://meme-suite.org/tools/meme, accessed on 21 May 2024). Motif number was set to 15, motif length was in the range of 6–200 amino acids, and default values were used for other parameters [45]. The structural information of the genes was obtained from the *A. membranaceus* genome annotation files, and the predicted results were visualized using the “Gene Structure View (Advanced)” tool of Tbtools (v1.108) [46]. For promoter analysis, the 2000 bp sequence upstream of the bZIP gene in the *A. membranaceus* genome was extracted and analyzed using PlantCARE (http://bioinformatics.psb.ugent.be/webtools/plantcare/html/, accessed on 22 May 2024) [47]. The predicted cis-acting elements were visualized with Tbtools (v1.108).

### 4.5. Chromosomal Localization of the bZIP Gene Family in A. membranaceus

Detailed chromosomal locations of all bZIP genes were localized to chromosomes based on gene annotation information provided by the *A. membranaceus* genome database in GPGD (http://www.gpgenome.com/species/109, accessed on 26 May 2024). Physical localization maps were drawn using the TBtools program (v1.108), which graphically displays the bZIP genes on *A. membranaceus* chromosomes.

### 4.6. Codon Usage Bias and Heterologous Expression Adaptability Analysis

The coding sequences (CDSs) of all identified AmbZIP genes were extracted from the *A. membranaceus* genome annotation files and subjected to codon usage analysis using CodonW 1.4.4. Several codon usage parameters, including GC content (GC, GC1, GC2, GC3, and GC3s), effective number of codons (ENC), codon adaptation index (CAI), codon bias index (CBI), frequency of optimal codons (FOP), and relative synonymous codon usage (RSCU), were calculated [48].

To investigate the factors influencing codon usage patterns, neutrality plot analysis was performed by plotting GC12 (the average of GC1 and GC2) against GC3, while ENC-GC3s plots were constructed to evaluate the relative contributions of mutation pressure and natural selection to codon usage variation. Parity Rule 2 (PR2) bias plots were generated based on the nucleotide composition at synonymous third codon positions [49].

Optimal codons were identified by comparing RSCU values between high- and low-bias gene groups. The CDSs were ranked according to ENC values, and the top 10% and bottom 10% of genes were selected as high- and low-bias groups, respectively. Codons with ΔRSCU > 0.08 and RSCU > 1 were defined as optimal codons [50].

To evaluate heterologous expression adaptability, codon usage frequency ratios (Sc/host) between AmbZIP genes and five representative hosts, including *Nicotiana tabacum*, *Arabidopsis thaliana*, *Solanum lycopersicum*, *Saccharomyces cerevisiae*, and *Escherichia coli*, were calculated according to previously reported methods [51]. Codons with Sc/host values ranging from 0.5 to 2.0 were considered adaptable, and the numbers of adaptable codons were compared among different hosts to assess codon compatibility and potential suitability for heterologous expression [52].

### 4.7. Analysis of Gene Duplication Events of the bZIP Members in A. membranaceus

The duplication events of the bZIP family genes in *A. membranaceus* were analyzed and visualized using MCScanX (https://chibba.agtec.uga.edu/mcscanx-web, accessed on 14 June 2024) [53]. The genomic data of *Z. mays*, *O. sativa*, *A. thaliana*, and *M. truncatula* were downloaded from NCBI GenBank database. The homologous genes of the bZIP genes in *Z. mays*, *O. sativa*, *A. thaliana* and *M. truncatula* genome were determined using MCScanX. KaKs_Calculator 2.0 software (https://sourceforge.net/projects/kakscalculator2/, accessed on 18 June 2024) was used to calculate the Ka and Ks of these duplication gene pairs, and the environmental selection pressure was further analyzed based on the ratio of Ka to Ks [54].

### 4.8. Gene Expression Analysis of the bZIP Members in A. membranaceus Based on Transcriptome Data

Based on the transcriptome functional annotation results, genes annotated as bZIP (basic leucine zipper) transcription factors were screened according to sequence similarity searches against the NR, Swiss-Prot, and Pfam databases. To ensure the reliability of annotation, the presence of the conserved bZIP domain (PF00170) was further verified using the Pfam database. The FPKM values [55] of the identified bZIP genes were extracted from the transcriptome dataset for each treatment group. Each treatment included three biological replicates, and the mean FPKM value of the three replicates was calculated to represent the final expression level of each gene under a given condition. To reduce the influence of large-scale expression differences among genes, the mean expression values were log_2_-transformed using the formula log_2_(FPKM + 1). Heatmaps were generated using the R package (version 1.0.13) pheatmap, with color gradients indicating the relative expression levels of genes across samples.

### 4.9. Quantitative Real-Time Fluorescence PCR Analysis

Total RNA was extracted from the *A. membranaceus* leaves with the Trizol reagent, according to the manufacturer’s instructions (Invitrogen, Waltham, MA, USA), and reverse transcription was conducted using a FastQuant RT Kit (TIANGEN, Beijing, China). qRT-PCR analysis was conducted using reported previously methods [6], and *actin8 gene* was used as the internal control. The cDNA obtained by reverse transcription was used as the template for qRT-PCR analysis. The qRT-PCR protocol was identical to the drought treatment used for transcriptome sequencing (20% PEG-6000 for 0, 6, 12, 24, and 72 h, with three biological replicates per time point). For qRT-PCR validation, 16 bZIP genes were selected from the transcriptome data based on the following criteria: (1) they exhibited significant differential expression (|log_2_FC| > 1, FDR < 0.05) under at least one drought treatment time point; (2) they covered different phylogenetic subgroups (including A, D, S, E, F, and I) to ensure representativeness; and (3) they displayed diverse expression trends (early induction, late induction, sustained upregulation, or suppression) to capture the functional heterogeneity of the AmbZIP family. Primers are shown in Table S6. To calculate amplification efficiency, a standard curve was generated from a 5-point 10-fold dilution series of cDNA for each primer pair. The amplification efficiency (E = 10^(−1/slope)^ − 1) of each primer pair ranged from 92% to 108%, and the R^2^ values were all above 0.99. Primers are shown in Table S6. The relative expression levels of the genes were determined using the 2^−∆∆Ct^ method.

### 4.10. Transcriptional Activation Activity and Subcellular Localization Analysis

One of the drought-induced bZIP genes, *AmbZIP46* was selected for further functional analysis. The transcriptional activation activity of *AmbZIP46* was analyzed with reference to the study of Wang et al. [56]. In brief, the recombinant plasmid PGBKT7-*AmbZIP46* was obtained by cloning the *AmbZIP46* gene into the pGBDT7 vector. The resulting recombinant plasmid and pGBKT7-empty (control) were separately transformed into the yeast strain AH109. The transformed strains of yeast were screened on single deficiency medium (-Trp) and double deficiency medium (-Trp/-His) with 4 different concentrations, cultured at 30 °C for 2~3 d and photographed. If the *AmbZIP46* protein has transcriptional activation activity, single colonies will grow on both single deficiency medium (-Trp) and double deficiency medium (-Trp/-His), otherwise they will not grow on the double deficiency medium (-Trp/-His).

For subcellular localization analysis, the PCR product of *AmbZIP46* and the vector pCAMBIA1305-GFP were double-cut with *Xba* I and *BamH* I, and then the T4 ligase was used to ligate the product and the vector. The resulting product was transformed into DH5α to identify positive single colonies. The vector pCAMBIA1305-GDP-*AmbZIP46* plasmid was transformed into the Agrobacterium strain GV3101 competent cells, injected into the lower epidermis of tobacco leaves (which were chosen for their high transient transformation efficiency, short experimental cycle, and optimal leaf transparency for confocal microscopy observation), and cultured for 2 d. The distribution of GFP fluorescence signals was observed under a laser confocal microscope (TCS SP8 X, Leica, Nussloch, Germany) to determine the location of the target protein.

## 5. Conclusions

In conclusion, this study systematically characterized the bZIP gene family in *A. membranaceus* through comprehensive analyses of genome-wide identification, phy-logeny, gene structure, conserved motifs, promoter cis-elements, gene duplication, co-don usage bias, expression profiling, and functional characterization. The results demonstrate that the AmbZIP family expanded predominantly through segmental du-plication under strong purifying selection and exhibits substantial functional diver-gence in promoter composition and drought-responsive expression patterns, suggesting the formation of a complex regulatory network involved in drought adaptation. Among the identified members, *AmbZIP46* was confirmed to possess transcriptional ac-tivation activity and nuclear localization, indicating that it is a promising candidate regulator in ABA-mediated drought responses. These findings provide valuable in-sights into the evolution and functional diversification of the bZIP gene family in *A. membranaceus* and establish a solid theoretical basis for future functional studies and molecular breeding aimed at improving drought tolerance. Future work involving transgenic validation and CRISPR/Cas-mediated gene editing will further clarify the biological functions and regulatory mechanisms of key AmbZIP genes.

## Data Availability

The datasets generated and/or analyzed during the current study are available in the NCBI Sequence Read Archive (SRA) repository (https://www.ncbi.nlm.nih.gov/sra/, accessed on 29 June 2024; BioProject: PRJNA1214058; SRA: SRR33154817-SRR33154831).

## Supplementary Materials

The following supporting information can be downloaded at: https://www.mdpi.com/article/10.3390/ijms27146275/s1.
