# Supplementary material for: Genome-Wide Characterization of bZIP Transcription Factors and Their Drought-Responsive Expression in Astragalus membranaceus
Source: Int J Mol Sci. 2026 Jul 14;27(14):6275. doi: 10.3390/ijms27146275 (PMC13410196; doi:10.3390/ijms27146275)
Supplement: Supplementary file 1 [file ijms-27-06275-s001.zip › ijms-4383784-supplementary.pdf]

Genome-wide characterization of bZIP transcription factors and their drought-responsive expression in *Astragalus membranaceus*

Jiemin Wang [1,2,†], Xiaoyuan Wang [1,2,†], Ye Zhang [1,2], Jiayao Chen [1,2], Lin Pei [3], Pei He [3], Huigai Sun [1,2,*] and Xiaowei Han [1,2,*]

1 College of Pharmacy, Hebei University of Chinese Medicine, Shijiazhuang 050200, China

2 Traditional Chinese Medicine Processing Technology Innovation Center of Hebei Province, Shijiazhuang, Hebei, China

3 Hebei Academy of Traditional Chinese Medicine, Shijiazhuang 050031, China

† These authors contributed equally to this work.

* Correspondence: sunhuigai66@163.com or sunhuigai@hebcm.edu.cn (H.S); hanxiaowei@hebcm.edu.cn or hanxiaowei2015 @126.com) (X.H).

Table S1. The physicochemical properties of bZIP family genes in *A. membranaceus*.

Gene name	Gene ID	Class	Number	MW (KDa)	pI	Instability index	Aliphatic index	Hydropathicity
AmbZIP1	Am01G006880	G	424	46.22	8.89	51.96	57.81	-0.90
AmbZIP2	Am01G010600	D	457	51.53	6.82	56.19	79.47	-0.58
AmbZIP3	Am01G011110	A	311	34.80	5.94	63.58	68.71	-0.84
AmbZIP4	Am01G017430	S	164	19.12	6.59	54.98	80.73	-0.89
AmbZIP5	Am01G017880	G	410	44.07	5.98	58.58	54.05	-0.91
AmbZIP6	Am01G019560	A	234	26.31	10.19	52.14	60.13	-0.88
AmbZIP7	Am01G021150	G	334	35.58	6.21	62.39	47.46	-0.94
AmbZIP8	Am01G021190	G	334	35.58	6.21	62.39	47.46	-0.94
AmbZIP9	Am01G023150	S	169	19.95	10.56	64.10	68.64	-0.99
AmbZIP10	Am01G029110	S	210	23.82	7.25	57.51	69.19	-0.91
AmbZIP11	Am01G037150	E	345	39.41	5.82	66.06	58.78	-1.14
AmbZIP12	Am01G039480	D	465	51.59	6.33	47.56	73.94	-0.50
AmbZIP13	Am01G039490	A	331	37.52	9.15	60.95	69.82	-0.82
AmbZIP14	Am01G042740	I	434	47.70	6.05	63.18	53.59	-0.90
AmbZIP15	Am02G000920	I	488	54.40	6.03	63.71	62.58	-0.86
AmbZIP16	Am02G001160	S	148	17.08	7.77	48.76	63.24	-0.69
AmbZIP17	Am02G003660	S	148	17.57	9.34	61.48	66.55	-0.89
AmbZIP18	Am02G007280	C	365	39.59	4.89	35.75	75.53	-0.46
AmbZIP19	Am02G021010	D	511	57.11	6.71	63.71	76.05	-0.56
AmbZIP20	Am02G022800	K	281	32.57	4.95	67.79	78.43	-0.46
AmbZIP21	Am02G023040	K	281	32.57	4.95	67.79	78.43	-0.46
AmbZIP22	Am02G026020	C	426	46.11	5.22	58.38	69.34	-0.66
AmbZIP23	Am02G028700	S	143	16.44	8.01	52.79	82.59	-0.68
AmbZIP24	Am02G031340	A	245	27.60	5.41	51.57	74.33	-0.70

AmbZIP25	Am02G038290	D	458	51.33	5.72	53.99	76.27	-0.57
AmbZIP26	Am03G000950	S	156	17.56	7.98	40.87	86.99	-0.58
AmbZIP27	Am03G002500	I	540	59.14	6.45	68.35	58.87	-0.91
AmbZIP28	Am03G002860	S	137	15.77	7.82	52.76	64.82	-0.92
AmbZIP29	Am03G004490	C	288	31.63	5.37	39.50	65.38	-0.66
AmbZIP30	Am03G007200	S	138	15.93	9.62	65.36	84.06	-0.66
AmbZIP31	Am03G007910	A	427	46.52	9.41	43.01	66.44	-0.69
AmbZIP32	Am03G011530	I	317	35.21	6.10	61.13	70.25	-0.68
AmbZIP33	Am03G015230	E	617	71.26	9.20	46.85	82.61	-0.62
AmbZIP34	Am03G021410	D	391	44.45	6.63	55.88	81.41	-0.41
AmbZIP35	Am03G025050	I	375	40.51	6.01	44.79	55.23	-0.79
AmbZIP36	Am03G030310	H	331	36.57	6.76	49.11	60.69	-0.95
AmbZIP37	Am03G032030	A	261	29.57	6.94	53.11	72.15	-0.86
AmbZIP38	Am03G034890	F	222	24.70	8.10	30.59	68.06	-0.75
AmbZIP39	Am03G035940	D	377	42.16	6.28	56.27	85.46	-0.57
AmbZIP40	Am03G036590	A	357	39.72	8.47	54.38	67.79	-0.86
AmbZIP41	Am04G004840	S	166	19.11	6.74	67.85	72.83	-0.72
AmbZIP42	Am04G010280	G	402	43.01	6.38	55.57	47.64	-0.96
AmbZIP43	Am04G016040	E	362	40.14	5.99	54.56	81.85	-0.58
AmbZIP44	Am04G016280	S	197	22.68	5.56	59.79	71.22	-0.79
AmbZIP45	Am04G017510	D	440	49.49	6.34	37.05	76.95	-0.61
AmbZIP46	Am04G018210	D	373	42.21	6.14	51.17	83.46	-0.47
AmbZIP47	Am04G018230	D	391	44.34	6.15	54.21	81.61	-0.51
AmbZIP48	Am05G013080	F	259	28.04	5.88	36.99	62.63	-0.67
AmbZIP49	Am05G013430	I	538	59.50	6.59	63.80	58.98	-0.96
AmbZIP50	Am05G013870	S	158	17.94	6.13	56.15	78.29	-0.58

AmbZIP51	Am05G019090	D	499	54.84	6.64	51.87	79.42	-0.43
AmbZIP52	Am05G021630	E	339	38.55	6.15	71.34	64.75	-1.01
AmbZIP53	Am05G024120	A	416	45.71	9.72	47.50	68.65	-0.80
AmbZIP54	Am05G028050	A	225	25.25	9.52	61.34	81.87	-0.79
AmbZIP55	Am05G028830	E	379	42.67	7.86	63.76	65.94	-0.92
AmbZIP56	Am05G029890	S	163	18.41	5.59	54.39	60.98	-0.79
AmbZIP57	Am05G037580	D	364	41.71	5.85	47.93	88.68	-0.37
AmbZIP58	Am06G022710	E	350	38.82	5.82	64.15	70.00	-0.73
AmbZIP59	Am07G004030	S	201	23.41	5.23	64.17	71.79	-0.92
AmbZIP60	Am07G006110	A	319	35.52	5.81	41.84	69.66	-0.80
AmbZIP61	Am07G008440	I	327	36.18	7.09	54.52	60.92	-0.83
AmbZIP62	Am07G016080	C	104	12.17	11.58	65.88	113.37	-0.38
AmbZIP63	Am08G008460	H	189	21.43	9.22	54.23	52.65	-1.27
AmbZIP64	Am08G008880	H	189	21.43	9.22	54.23	52.65	-1.27
AmbZIP65	Am09G008470	S	198	22.96	6.44	80.02	67.88	-0.90
AmbZIP66	Am09G010420	A	325	35.82	7.90	60.70	66.31	-0.79
AmbZIP67	Am09G011020	B	798	86.96	5.69	49.24	65.93	-0.59
AmbZIP68	Am09G011360	D	461	51.06	7.13	57.61	72.30	-0.57
AmbZIP69	Am09G012710	D	486	55.00	6.90	58.46	70.51	-0.66
AmbZIP70	Am09G018040	J	492	54.86	8.57	76.50	65.87	-0.83
AmbZIP71	Am09G018150	A	417	46.06	8.80	65.47	63.79	-0.78
AmbZIP72	Am09G020270	S	147	16.66	6.32	55.02	92.99	-0.55
AmbZIP73	Am09G022920	C	417	44.94	6.78	56.29	61.77	-0.74
AmbZIP74	Am09G023630	G	420	44.80	7.22	51.91	56.10	-0.83

Table S2. Ka/Ks values of paralogous bZIP gene pairs in *A. membranaceus*.

Gene pair	Ka	Ks	Ka/Ks
AmbZIP2-AmbZIP39	0.10	0.70	0.15
AmbZIP3-AmbZIP40	0.19	1.29	0.15
AmbZIP9-AmbZIP30	0.57	4.56	0.12
AmbZIP5-AmbZIP42	0.10	0.60	0.17
AmbZIP9-AmbZIP41	0.19	0.96	0.20
AmbZIP11-AmbZIP52	0.18	0.85	0.22
AmbZIP12-AmbZIP51	0.15	0.54	0.28
AmbZIP11-AmbZIP58	0.32	NaN	NaN
AmbZIP10-AmbZIP65	0.63	3.01	0.21
AmbZIP13-AmbZIP71	0.44	2.34	0.19
AmbZIP2-AmbZIP68	0.20	3.95	0.05
AmbZIP15-AmbZIP27	0.21	0.82	0.25
AmbZIP16-AmbZIP28	0.32	1.94	0.17
AmbZIP16-AmbZIP26	0.40	2.91	0.14
AmbZIP17-AmbZIP30	0.27	0.96	0.28
AmbZIP18-AmbZIP29	0.23	0.89	0.26
AmbZIP22-AmbZIP73	0.21	0.72	0.28
AmbZIP23-AmbZIP72	0.15	1.08	0.14
AmbZIP19-AmbZIP69	0.11	0.51	0.22
AmbZIP26-AmbZIP28	0.39	NaN	NaN
AmbZIP33-AmbZIP46	0.20	0.83	0.24
AmbZIP30-AmbZIP41	0.50	2.54	0.20
AmbZIP26-AmbZIP50	0.22	0.98	0.22
AmbZIP31-AmbZIP53	0.33	2.04	0.16

AmbZIP27-AmbZIP49	0.40	2.90	0.14
AmbZIP28-AmbZIP50	0.38	3.50	0.11
AmbZIP33-AmbZIP57	0.42	1.80	0.23
AmbZIP29-AmbZIP62	0.30	NaN	NaN
AmbZIP46-AmbZIP57	0.42	3.42	0.12
AmbZIP44-AmbZIP65	0.35	1.79	0.20
AmbZIP52-AmbZIP58	0.29	2.52	0.12
AmbZIP59-AmbZIP65	0.20	1.09	0.18
AmbZIP63-AmbZIP64	0.00	0.00	NaN

*NaN: Not a number

Table S3. Analysis of nucleotide composition and codon usage bias of bZIP genes in *A. membranaceus*.

Gene name	Gene ID	GC_first	GC_second	GC_third	GC_total	GC3s	Nc	CAI	CBI	Fop
AmbZIP1	Am01G006880	50.59	48.24	33.88	44.24	0.32	48.47	0.20	-0.07	0.39
AmbZIP2	Am01G010600	53.49	42.36	40.61	45.49	0.38	56.19	0.18	-0.06	0.39
AmbZIP3	Am01G011110	54.17	41.99	36.86	44.34	0.34	51.67	0.18	-0.14	0.34
AmbZIP4	Am01G017430	50.30	30.91	35.15	38.79	0.33	45.43	0.19	-0.02	0.41
AmbZIP5	Am01G017880	56.93	49.64	34.79	47.12	0.32	51.87	0.19	-0.11	0.37
AmbZIP6	Am01G019560	38.3	45.53	45.11	42.98	0.44	50.83	0.18	-0.05	0.41
AmbZIP7	Am01G021150	54.93	51.94	33.13	46.67	0.30	48.51	0.20	-0.11	0.38
AmbZIP8	Am01G021190	54.93	51.94	33.43	46.77	0.30	48.63	0.20	-0.11	0.38
AmbZIP9	Am01G023150	51.18	44.12	52.35	49.22	0.51	55.42	0.18	0.13	0.48
AmbZIP10	Am01G029110	50.71	35.55	35.55	40.60	0.34	50.65	0.22	-0.04	0.40
AmbZIP11	Am01G037150	51.45	35.84	35.84	41.04	0.34	54.14	0.19	-0.18	0.33
AmbZIP12	Am01G039480	54.51	43.99	38.20	45.57	0.35	53.09	0.17	-0.13	0.35
AmbZIP13	Am01G039490	49.70	39.46	31.02	40.06	0.29	50.70	0.17	-0.18	0.33
AmbZIP14	Am01G042740	55.86	42.99	38.16	45.67	0.35	51.35	0.20	-0.12	0.37
AmbZIP15	Am02G000920	49.90	40.49	35.99	42.13	0.34	50.22	0.21	-0.07	0.39
AmbZIP16	Am02G001160	38.26	33.56	40.27	37.36	0.34	43.07	0.17	-0.21	0.32
AmbZIP17	Am02G003660	46.31	34.90	59.73	46.98	0.56	48.49	0.14	-0.08	0.37
AmbZIP18	Am02G007280	50.82	43.44	39.62	44.63	0.38	54.60	0.23	0.00	0.42
AmbZIP19	Am02G021010	53.91	41.99	33.98	43.29	0.31	49.55	0.18	-0.15	0.34
AmbZIP20	Am02G022800	51.77	37.23	54.26	47.75	0.52	49.59	0.21	0.06	0.44
AmbZIP21	Am02G023040	51.77	37.59	54.26	47.87	0.52	49.87	0.21	0.07	0.45
AmbZIP22	Am02G026020	57.38	46.37	40.28	48.01	0.38	52.28	0.22	0.07	0.46
AmbZIP23	Am02G028700	55.56	39.58	48.61	47.92	0.46	53.59	0.19	0.00	0.41
AmbZIP24	Am02G031340	56.10	40.65	58.54	51.76	0.56	53.08	0.18	0.03	0.43

AmbZIP25	Am02G038290	55.34	39.22	35.95	43.50	0.33	53.68	0.19	-0.06	0.39
AmbZIP26	Am03G000950	46.50	40.13	41.40	42.68	0.40	43.46	0.17	-0.05	0.39
AmbZIP27	Am03G002500	51.76	44.55	34.75	43.68	0.33	48.40	0.17	-0.13	0.35
AmbZIP28	Am03G002860	39.13	38.41	41.3	39.61	0.38	43.98	0.19	-0.17	0.35
AmbZIP29	Am03G004490	50.87	42.91	36.33	43.37	0.35	53.84	0.20	-0.07	0.39
AmbZIP30	Am03G007200	49.64	38.85	46.76	45.08	0.44	52.23	0.17	0.08	0.46
AmbZIP31	Am03G007910	52.80	40.89	38.08	43.93	0.35	44.87	0.18	-0.10	0.37
AmbZIP32	Am03G011530	54.40	42.14	44.34	46.96	0.43	53.93	0.20	0.01	0.43
AmbZIP33	Am03G015230	54.53	38.67	37.38	43.53	0.34	53.61	0.19	-0.05	0.39
AmbZIP34	Am03G021410	51.53	39.29	34.95	41.92	0.30	49.41	0.18	-0.08	0.38
AmbZIP35	Am03G025050	53.46	47.87	52.66	51.33	0.50	60.96	0.20	-0.04	0.42
AmbZIP36	Am03G030310	50.30	45.18	34.04	43.17	0.33	50.28	0.17	-0.18	0.33
AmbZIP37	Am03G032030	49.24	43.13	42.37	44.91	0.40	48.65	0.13	-0.23	0.29
AmbZIP38	Am03G034890	44.84	40.81	27.35	37.67	0.26	41.44	0.19	-0.13	0.36
AmbZIP39	Am03G035940	54.76	41.27	38.62	44.89	0.36	51.27	0.16	-0.13	0.35
AmbZIP40	Am03G036590	59.22	41.06	44.41	48.23	0.42	55.87	0.22	0.01	0.43
AmbZIP41	Am04G004840	47.90	42.51	53.29	47.90	0.52	54.70	0.18	0.07	0.46
AmbZIP42	Am04G010280	54.84	51.61	34.24	46.90	0.31	53.26	0.19	-0.08	0.39
AmbZIP43	Am04G016040	53.44	38.84	40.22	44.17	0.39	53.36	0.19	-0.08	0.37
AmbZIP44	Am04G016280	48.99	34.34	32.83	38.72	0.30	46.07	0.19	-0.08	0.38
AmbZIP45	Am04G017510	55.78	39.68	36.73	44.07	0.34	52.01	0.20	-0.11	0.37
AmbZIP46	Am04G018210	51.34	38.77	32.89	41.00	0.30	50.37	0.18	-0.09	0.37
AmbZIP47	Am04G018230	51.28	38.27	33.67	41.07	0.30	51.35	0.18	-0.10	0.36
AmbZIP48	Am05G013080	55.38	45.00	41.15	47.18	0.40	54.54	0.23	-0.02	0.42
AmbZIP49	Am05G013430	52.69	43.04	35.81	43.85	0.33	50.99	0.19	-0.07	0.39
AmbZIP50	Am05G013870	42.14	36.48	42.14	40.25	0.39	46.92	0.17	-0.03	0.41

AmbZIP51	Am05G019090	56.00	45.00	33.80	44.93	0.32	47.91	0.16	-0.13	0.35
AmbZIP52	Am05G021630	51.18	38.24	43.24	44.22	0.41	53.08	0.18	-0.14	0.35
AmbZIP53	Am05G024120	50.12	43.41	30.22	41.25	0.27	44.08	0.15	-0.12	0.35
AmbZIP54	Am05G028050	52.65	42.92	42.04	45.87	0.41	45.81	0.24	0.17	0.51
AmbZIP55	Am05G028830	53.42	41.58	35.79	43.6	0.34	50.11	0.2	-0.1	0.37
AmbZIP56	Am05G029890	41.46	35.37	40.24	39.02	0.36	46.71	0.23	0.02	0.45
AmbZIP57	Am05G037580	50.41	35.34	30.41	38.72	0.27	49.22	0.19	-0.1	0.36
AmbZIP58	Am06G022710	51.57	43.02	40.74	45.11	0.39	55.24	0.2	-0.06	0.39
AmbZIP59	Am07G004030	43.07	33.17	30.2	35.48	0.26	44.06	0.17	-0.14	0.34
AmbZIP60	Am07G006110	54.06	38.75	38.75	43.85	0.36	46.15	0.19	-0.09	0.37
AmbZIP61	Am07G008440	55.49	44.21	48.78	49.49	0.47	51.89	0.19	-0.01	0.42
AmbZIP62	Am07G016080	49.52	37.14	40.95	42.54	0.39	42.09	0.12	-0.11	0.31
AmbZIP63	Am08G008460	43.68	43.16	41.05	42.63	0.38	48.82	0.19	-0.03	0.41
AmbZIP64	Am08G008880	43.68	43.16	41.05	42.63	0.38	48.82	0.19	-0.03	0.41
AmbZIP65	Am09G008470	46.23	36.18	36.68	39.7	0.34	46.82	0.17	-0.15	0.34
AmbZIP66	Am09G010420	56.75	43.25	35.58	45.19	0.32	47.82	0.18	-0.09	0.37
AmbZIP67	Am09G011020	53.19	43.68	41.43	46.1	0.39	47.89	0.22	-0.05	0.4
AmbZIP68	Am09G011360	52.16	44.81	38.53	45.17	0.35	54.33	0.17	-0.1	0.38
AmbZIP69	Am09G012710	55.44	40.66	37.17	44.42	0.33	51.89	0.18	-0.16	0.33
AmbZIP70	Am09G018040	48.28	46.25	36.11	43.54	0.35	54.78	0.19	-0.08	0.38
AmbZIP71	Am09G018150	54.78	42.11	36.36	44.42	0.33	43.11	0.17	-0.14	0.35
AmbZIP72	Am09G020270	54.05	39.19	48.65	47.3	0.46	59.23	0.19	0.04	0.44
AmbZIP73	Am09G022920	50.48	47.37	32.78	43.54	0.31	47.37	0.18	-0.14	0.36
AmbZIP74	Am09G023630	54.63	51.78	31.59	46	0.29	46.08	0.18	-0.12	0.36

Table S4. Optimal codon usage statistics of the *A. membranaceus* bZIP gene family.

amino acids	Codon	High-expression group		Low-expression group		ΔRSCU
		Number	RSCU	Number	RSCU	
Ala	**GCT***	54	2.16	62	1.43	0.73
	GCC	6	0.24	39	0.9	-0.66
	**GCA***	35	1.4	49	1.13	0.27
	GCG	5	0.2	23	0.53	-0.33
Tyr	TAT	23	1.24	22	1.22	0.02
	TAC	14	0.76	14	0.78	-0.02
His	**CAT***	32	1.31	33	1.1	0.21
	CAC	17	0.69	27	0.9	-0.21
Gln	**CAA***	90	1.36	105	1.17	0.2
	CAG	42	0.64	75	0.83	-0.2
Asn	**AAT***	95	1.19	76	1.03	0.15
	AAC	65	0.81	71	0.97	-0.15
Lys	AAA	42	0.99	58	0.94	0.05
	AAG	43	1.01	65	1.06	-0.05
Asp	**GAT***	57	1.65	70	1.12	0.53
Phe	**TTT***	27	1.5	38	1.09	0.41
	TTC	9	0.5	32	0.91	-0.41
Leu	**TTA***	40	1.45	36	0.96	0.49
	TTG	38	1.38	51	1.37	0.02
	**CTT***	37	1.35	47	1.26	0.09
	CTC	20	0.73	35	0.94	-0.21
	CTA	14	0.51	28	0.75	-0.24
	CTG	16	0.58	27	0.72	-0.14

Ile	ATT	29	1.43	51	1.38	0.05
	ATC	16	0.79	32	0.86	-0.08
	ATA	16	0.79	28	0.76	0.03
Val	GTT	42	1.62	40	1.68	-0.07
	GTC	6	0.23	14	0.59	-0.36
	GTA	15	0.58	19	0.8	-0.22
	**GTG***	41	1.58	22	0.93	0.65
	GAC	12	0.35	55	0.88	-0.53
Glu	**GAA***	75	1.26	94	1.09	0.17
	GAG	44	0.74	79	0.91	-0.17
Cys	**TGT***	19	1.81	12	1.09	0.72
	TGC	2	0.19	10	0.91	-0.72
Arg	CGT	7	0.33	27	0.92	-0.58
	CGC	1	0.05	20	0.68	-0.63
	CGA	5	0.24	25	0.85	-0.61
	CGG	6	0.28	27	0.92	-0.63
	**AGA***	61	2.88	29	0.98	1.9
	**AGG***	47	2.22	49	1.66	0.56
Gly	**GGT***	44	1.66	50	1.56	0.1
	GGC	4	0.15	27	0.84	-0.69
	**GGA***	37	1.4	34	1.06	0.33
	GGG	21	0.79	17	0.53	0.26
Ser	**TCT***	49	1.7	66	1.47	0.23
	TCC	9	0.31	43	0.96	-0.65
	**TCA***	57	1.98	64	1.43	0.55
	TCG	11	0.38	32	0.71	-0.33

	**AGT***	32	1.11	41	0.91	0.2
	AGC	15	0.52	23	0.51	0.01
Pro	**CCT***	33	1.74	51	1.32	0.41
	CCC	4	0.21	24	0.62	-0.41
	**CCA***	25	1.32	46	1.19	0.12
	CCG	14	0.74	33	0.86	-0.12
Thr	**ACT***	36	1.44	46	1.22	0.22
	ACC	8	0.32	36	0.95	-0.63
	**ACA***	50	2	52	1.38	0.62
	ACG	6	0.24	17	0.45	-0.21

*and bold indicate high-expression and optimal codons, respectively.

Table S5. Codon usage frequencies and ratios in *A. membranaceus* and five heterologous hosts.

Codon	*A. membranaceus* bZIP	*N. tabacum*	*A. thaliana*	*S. lycopersicum*	*E. coli*	*S. cerevisiae*	Am/ *N. tabacum*	Am/ *A. thaliana*	Am/ *S. lycopersicum*	Am/ *E. coli*	Am/ *S. cerevisiae*
AAA	24.031	32.6	30.8	31.1	37.2	41.9	0.74	0.78	0.77	0.65	0.57
AAC	22.407	17.9	20.9	17.3	20.3	24.8	1.25	1.07	1.30	1.10	0.90
AAG	27.441	33.5	32.7	31	15.3	30.8	0.82	0.84	0.89	1.79	0.89
AAU	37.426	28	22.3	30.5	29.3	35.7	1.34	1.68	1.23	1.28	1.05
ACA	17.942	17.4	15.7	17.9	15.1	17.8	1.03	1.14	1.00	1.19	1.01
ACC	8.93	9.7	10.3	8.6	18.9	12.7	0.92	0.87	1.04	0.47	0.70
ACG	3.978	4.5	7.7	4.6	13.6	8	0.88	0.52	0.86	0.29	0.50
ACU	20.012	20.3	17.5	19.9	13.1	20.3	0.99	1.14	1.01	1.53	0.99
AGA	19.931	16	24.2	25.6	7.1	21.3	1.25	0.82	0.78	2.81	0.94
AGC	9.296	10	9.2	9.7	14.3	9.8	0.93	1.01	0.96	0.65	0.95
AGG	18.104	12.2	10.2	10.8	4	9.2	1.48	1.77	1.68	4.53	1.97
AGU	18.186	13.3	22.2	23.9	13.2	14.2	1.37	0.82	0.76	1.38	1.28
AUA	9.986	14	12.6	14	13.3	17.8	0.71	0.79	0.71	0.75	0.56
AUC	8.606	13.9	18.5	14	19.4	17.2	0.62	0.47	0.61	0.44	0.50
AUG	32.393	25	24.5	24.7	23.7	20.9	1.30	1.32	1.31	1.37	1.55
AUU	17.09	27.8	21.5	28.2	29.6	30.1	0.61	0.79	0.61	0.58	0.57
CAA	40.309	20.7	19.4	21	14.4	27.3	1.95	2.08	1.92	2.80	1.48
CAC	8.646	8.7	8.7	7.8	7.3	7.8	0.99	0.99	1.11	1.18	1.11
CAG	28.05	15	15.2	14	26.7	12.1	1.87	1.85	2.00	1.05	2.32
CAU	17.78	13.4	13.8	15.5	12.4	13.6	1.33	1.29	1.15	1.43	1.31
CCA	18.104	19.8	16.1	19.2	9.1	18.3	0.91	1.12	0.94	1.99	0.99
CCC	7.144	6.6	5.3	5.7	6.2	6.8	1.08	1.35	1.25	1.15	1.05
CCG	6.292	5	8.6	4.6	14.5	5.3	1.26	0.73	1.37	0.43	1.19

CCU	22.61	18.7	18.7	19.2	9.5	13.5	1.21	1.21	1.18	2.38	1.67
CGA	7.185	5.3	19	16.4	4.8	3	1.36	0.38	0.44	1.50	2.40
CGC	5.236	3.9	11.3	9.3	14	2.6	1.34	0.46	0.56	0.37	2.01
CGG	5.683	3.7	11	11.9	7.9	1.7	1.54	0.52	0.48	0.72	3.34
CGU	8.037	7.5	14	15.2	15.9	6.4	1.07	0.57	0.53	0.51	1.26
CUA	8.89	9.4	9.9	10	5.6	13.4	0.95	0.90	0.89	1.59	0.66
CUC	11.488	12.3	16.1	11.2	9.5	5.4	0.93	0.71	1.03	1.21	2.13
CUG	10.635	10.2	9.8	10.5	37.4	10.5	1.04	1.09	1.01	0.28	1.01
CUU	22.732	24	24.1	24.9	14.5	12.3	0.95	0.94	0.91	1.57	1.85
GAA	38.482	36	34.3	34.8	35.1	45.6	1.07	1.12	1.11	1.10	0.84
GAC	13.883	16.9	17.2	15	17.9	20.2	0.82	0.81	0.93	0.78	0.69
GAG	32.109	29.4	32.2	26.6	19.4	19.2	1.09	1.00	1.21	1.66	1.67
GAU	36.249	36.9	36.6	39.3	33.7	37.6	0.98	0.99	0.92	1.08	0.96
GCA	20.865	23.1	17.5	22.2	23	16.2	0.90	1.19	0.94	0.91	1.29
GCC	10.026	12.5	10.3	10.1	21.6	12.6	0.80	0.97	0.99	0.46	0.80
GCG	5.48	5.8	9	5.2	21.1	6.2	0.94	0.61	1.05	0.26	0.88
GCU	30.485	31.2	28.3	30.7	18.9	21.2	0.98	1.08	0.99	1.61	1.44
GGA	20.296	23.2	1.2	0.9	13.6	10.9	0.87	16.91	22.55	1.49	1.86
GGC	7.794	11.2	7.2	6.7	20.6	9.8	0.70	1.08	1.16	0.38	0.80
GGG	9.783	10.5	12.5	13.5	12.3	6	0.93	0.78	0.72	0.80	1.63
GGU	21.433	22.3	10.5	10.8	23.7	23.9	0.96	2.04	1.98	0.90	0.90
GUA	6.901	11.4	9.9	11.2	13.1	11.8	0.61	0.70	0.62	0.53	0.58
GUC	6.129	11.1	12.8	10.1	13.1	11.8	0.55	0.48	0.61	0.47	0.52
GUG	12.827	16.7	17.4	16	19.9	10.8	0.77	0.74	0.80	0.64	1.19
GUU	20.499	26.8	27.2	28	21.6	22.1	0.76	0.75	0.73	0.95	0.93
UAA	0.69	1.1	0.9	0.9	2	1.1	0.63	0.77	0.77	0.35	0.63

UAC	6.292	13.5	13.7	12.4	11.7	14.8	0.47	0.46	0.51	0.54	0.43
UAG	0.812	0.5	0.5	0.5	0.3	0.5	1.62	1.62	1.62	2.71	1.62
UAU	11.731	17.8	14.6	18.6	21.6	18.8	0.66	0.80	0.63	0.54	0.62
UCA	27.4	17.6	18.3	20.7	13.1	18.7	1.56	1.50	1.32	2.09	1.47
UCC	13.233	10.2	11.2	9.9	9.7	14.2	1.30	1.18	1.34	1.36	0.93
UCG	8.727	5.3	9.3	5.6	8.2	8.6	1.65	0.94	1.56	1.06	1.01
UCU	31.906	20	25.2	21.2	13.1	23.5	1.60	1.27	1.51	2.44	1.36
UGA	1.502	1	6.3	5.4	1.1	0.7	1.50	0.24	0.28	1.37	2.15
UGC	3.044	7.2	3.8	3.1	5.5	4.8	0.42	0.80	0.98	0.55	0.63
UGG	7.713	12.2	4.9	3.1	13.4	10.4	0.63	1.57	2.49	0.58	0.74
UGU	6.17	9.8	9	6.9	5.9	8.1	0.63	0.69	0.89	1.05	0.76
UUA	14.573	13.4	12.7	14.4	17.4	26.2	1.09	1.15	1.01	0.84	0.56
UUC	11.325	18	20.7	17.5	13.9	18.4	0.63	0.55	0.65	0.81	0.62
UUG	20.54	22.3	20.9	24.2	12.9	27.2	0.92	0.98	0.85	1.59	0.76
UUU	16.521	25.1	21.8	26	24.4	26.1	0.66	0.76	0.64	0.68	0.63

Table S6. The sequences of primers used in qRT-PCR analysis.

Gene name	Forward primer (5′-3′)	Reverse primer (5′-3′)	Efficiency (%)
AmbZIP72	GGTACAATCGACGACAGG	TAGCGGCTTCGGTTTCA	96.3
AmbZIP56	CAGCACGTAGATCAAGGA	CCATCTGAACCCTAAGAATA	98.1
AmbZIP57	TGAGCCAGAACATAGGAG	GCAGAAGCCAAGTCATTAG	95.7
AmbZIP38	GTGAGGAAACTACAAGGGAA	GATGAAAGCATGGCAAAT	102.4
AmbZIP50	TCTCGGATGAGGAAACAG	CATGTAGGACAAAGGGTTG	94.8
AmbZIP41	TCTGCTATTCCCTCTTCCG	TCATTCGCCTCAGCTTTC	97.2
AmbZIP28	TCTCGGATGAGAAAGCAG	AGCCTCCACATTCAAATAG	99.5
AmbZIP14	CGGTTACTCACCTACTGGAA	GCACGCCTATGACCTCTGT	93.6
AmbZIP34	TTTCTTGTGGATTGGTGGTT	TTTCCTGCCGTAGATGGT	96.0
AmbZIP22	CATCACGACTCCTCCAACA	GATATTCATCCGAATCAACG	101.5
AmbZIP18	CGGTAGTGATAATCAAGGTAAG	GTTGGCTGGCATCTGTAA	95.2
AmbZIP54	GGCGTCGTCTAAGGTTGT	GGTTGAGGGTTTGGTGTC	97.9
AmbZIP5	CAGCTACGTCACAGGAGCAA	CGAAGCATCCGCAATACC	98.6
AmbZIP46	TGGATCGGAGGATCTCGT	GCAGCAGCCATCTGAAAC	104.3
AmbZIP60	ATTCAATACCAACTCCCTTCA	CGAGCAGCAGATTCCCTA	92.7
AmbZIP37	AGAGGAGGCTGAGGAGGA	CAATGAATCTGGCGGTAA	96.8
AmActin8	TTATTGGGCGACCACGAC	TGTTGGCTTTGGGATTGA	99.2
